# Reader Architectures for Wireless Surface Acoustic Wave Sensors

**DOI:** 10.3390/s18061734

**Published:** 2018-05-28

**Authors:** Fabian Lurz, Thomas Ostertag, Benedict Scheiner, Robert Weigel, Alexander Koelpin

**Affiliations:** 1 Institute for Electronics Engineering, Friedrich-Alexander University Erlangen-Nuremberg (FAU), 91058 Erlangen, Germany; benedict.scheiner@fau.de (B.S.); robert.weigel@fau.de (R.W.); 2 RSSI GmbH, Bürgermeister-Graf-Ring 1, 82538 Geretsried, Germany; thomas.ostertag@rssi.de; 3 Department of General Electrical Engineering and Measurement Technology, Brandenburg University of Technology, 03046 Cottbus, Germany; alexander.koelpin@btu.de

**Keywords:** surface acoustic waves, transceiver architecture, temperature sensor, pressure sensor, torque sensor, wireless sensor, frequency measurement

## Abstract

Wireless surface acoustic wave (SAW) sensors have some unique features that make them promising for industrial metrology. Their decisive advantage lies in their purely passive operation and the wireless readout capability allowing the installation also at particularly inaccessible locations. Furthermore, they are small, low-cost and rugged components on highly stable substrate materials and thus particularly suited for harsh environments. Nevertheless, a sensor itself does not carry out any measurement but always requires a suitable excitation and interrogation circuit: a reader. A variety of different architectures have been presented and investigated up to now. This review paper gives a comprehensive survey of the present state of reader architectures such as time domain sampling (TDS), frequency domain sampling (FDS) and hybrid concepts for both SAW resonators and reflective SAW delay line sensors. Furthermore, critical performance parameters such as measurement accuracy, dynamic range, update rate, and hardware costs of the state of the art in science and industry are presented, compared and discussed.

## 1. Introduction

The first ideas of using surface acoustic wave (SAW) devices as sensors have already been developed around 40 years ago [1,2,3,4] with a continuous development since then for a wide range of application fields. SAW sensors are proven to work in harsh environments [5] and are used for sensing of temperature, pressure, torque, acceleration, humidity and more [6,7,8,9,10,11,12,13,14,15,16,17,18,19,20].

Compared to today’s booming and high-volume SAW and bulk acoustic wave (BAW) filters with an estimated more than 40 billion radio frequency (RF) acoustic filter functions implemented in mobile phones in 2015 [21], SAW-based wireless sensing is still a low-volume professional niche application. However, this might change in the future: pushed by the fourth industrial revolution, sensor technology for professional metrology applications plays an increasingly important role. In particular, the interest in wireless solutions is growing, as a fixed wired connection cannot be established in most use-cases. The use of slip rings and brushes is usually also undesirable because they cause mechanical and electrical problems or, in the case of RF signal transmission, they are difficult to impossible to implement. For this purpose, radio sensors with wireless interrogation must be used. SAW sensors have some unique features that make them promising for such application scenarios: their decisive advantage lies in their purely passive operation and the wireless readout capability allowing the installation at particularly inaccessible locations. Furthermore, they are small, low-cost and rugged components on highly stable substrate materials and thus particularly suited for harsh environments. Nevertheless, a sensor itself does not carry out any measurement but always requires a suitable excitation and interrogation circuit: a reader. Despite the wide range of possible applications in industrial and automotive sensing, the reader is still a bottleneck today, especially in terms of cost, which prevents wireless SAW sensors from being employed on a larger scale.

This review paper provides a comprehensive overview of the current state of the art of wireless SAW readers and discusses the advantages and disadvantages of currently used architectures. Section 2 will give an introduction to the fundamental concept of wireless SAW sensing with the two sensor principles: reflective delay lines and resonant SAWs. Section 3 will categorize the SAW readers architectures according to their sensor type and readout concept (time domain sampling, frequency domain sampling and hybrid approaches). The individual architectures are then introduced and discussed in detail in Section 4 and Section 5. Finally, Section 6 is dedicated to an overall comparison and discussion of all reader architectures and Section 7 concludes this paper.

## 2. Fundamental Concept of Passive SAW Sensors

**Figure 1:**
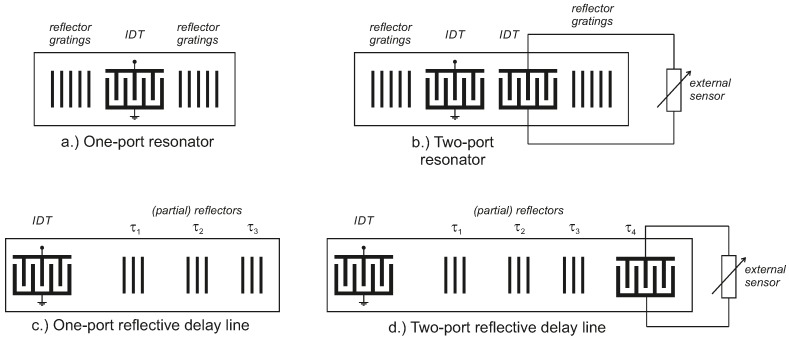
Schematic layout of passive surface acoustic wave (SAW) devices for sensor applications.

The fundamental concept of passive SAW sensors is based on one or two interdigital transducer (IDTs) [22] arranged on the surface of a piezoelectric substrate. With the IDT being connected to an antenna, it transforms the received electromagnetic signal into a surface acoustic wave and vice versa [13]. There are two different principles of SAW structures used for sensing: the first type is based on SAW resonators, whose resonance frequency is influenced by the external measurand. One-port resonators are directly affected by, e.g., temperature, torque while two-port devices are typically electrically loaded by a conventional sensor [23,24]. It should be noted, however, that these impedance loaded sensors only produce minimal effects that are difficult for the reader to detect. Furthermore, the passive load is probed in the RF range instead of with direct current (DC) and so the characteristic of the SAW sensor can be significantly different from the expected DC characteristic. The second type are delay line structures where the measurand either affects the velocity $v_{\mathrm{saw}}$ of the surface wave or the geometrical length *L* of the propagation path both yielding to a different round trip delay $\tau_{x}=L/v_{\mathrm{saw}}$ of the readout signals. Due to the (relatively) low propagation velocity of the SAW with, depending on the used substrate up to $10^{5}$ times smaller than electromagnetic waves (in air), relatively long delays can be realized at compact size [14]. Reflective delay SAWs can also be impedance loaded with external sensors with the same challenges as the SAW resonators. Figure 1 shows the schematic layout of SAW one-port and two-port resonators as well as reflective delay line sensors.

SAW reflective delay lines were originally proposed in 1972 [25] for wireless identification systems (“ID-Tags”) and are still used today in applications where CMOS-based radio frequency identification (RFID) systems reach their limits [26,27]. It took a while until reflective delay line SAWs were first used as wireless temperature sensors in 1987 [28]. An important advantage is that they can combine both identification and wireless sensing [29]. Modern systems even provide multiple access capabilities by using time division multiple access (TDMA) [30], code division multiple access (CDMA) [31,32,33] or orthogonal frequency coding (OFC) [34]. One-port reflective delay line sensors use a single IDT together with several partial reflectors, which leads to several delayed responses of the interrogation signal. Two-port or three-port reflective delay lines use multiple IDTs of which one or two are impedance-loaded by an external sensor [35,36]. A relative evaluation of these response signals with respect to each other results in the sensor value.

Resonant SAW sensors work slightly differently because the sensor value is represented as a frequency. A one-port resonant SAW sensor has a single IDT in the center between two reflector gratings that are forming a resonant cavity for the surface wave with a resonance frequency $f_{0}$ [7]. Hence, the pitch between the fingers of the reflecting grating define the resonance frequency of the SAW. When an external influence (like force and/or temperature) affects the sensor, its resonance frequency changes. A two-port resonator typically connects one IDT to the antenna, for RF signal reception and transmission, and the second IDT to an external sensor that loads the resonator, depending on the external measurand. SAW resonators have the advantage that they possess a lower insertion loss compared to reflective delay lines [37]. Furthermore, their high quality factor ($Q_{SAW}$) potentially ensures a higher resolution [38,39]. However, resonant sensors can only employ frequency division multiple access (FDMA) and not TDMA, CDMA or OFC like reflective delay lines.

In almost all of today’s systems, not only is a single SAW used, but several in a certain configuration to increase the robustness as well as the sensitivity of the system. The idea to use two resonators with a differential evaluation was first introduced in [40] and further discussed in [41]. This has the significant advantage that disturbing influences that affect both SAWs to the same extent, e.g., impedance changes in the radio channel, are canceled out. For highly accurate strain, torque and/or pressure measurements, typically even three or more resonators are used: two for a differential sensing of the measurand and a third one, mechanically unloaded, for temperature compensation [42].

## 3. SAW Reader Architecture Classification

**Figure 2:**
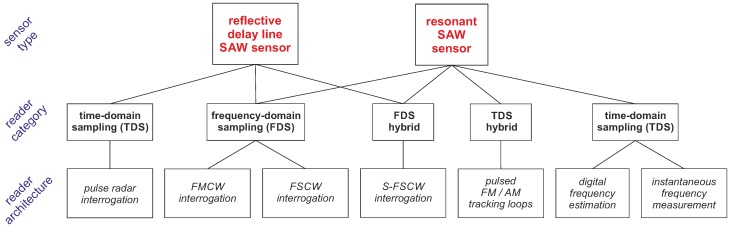
Sensor and current reader architectures with their classification for wireless SAW instrumentation.

As general partitioning, all architectures are currently divided into two categories: time domain sampling (TDS), also known as wideband or full band sampling, and frequency domain sampling (FDS), also known as narrowband or partial band sampling as proposed by Pohl in 2000 [23]. Herewith, we suggest a further subdivision into FDS/TDS hybrid concepts to consider recent developments such as switched frequency stepped continuous wave (S-FSCW) and pulsed frequency modulation (FM)/amplitude modulation (AM) tracking loops. As will be shown in detail later on, both architectures have influences from TDS as well as FDS with partly mixed approaches in the concept, front end and signal processing. Figure 2 provides an overview of the different sensor types, reader architectures and the proposed classification in different reader categories. Both SAW sensor types (reflective delay line and resonant SAWs) can be interrogated either way (TDS/FDS), but not necessarily with each reader architecture. As each sensor type and reader architecture has its distinctive advantages and disadvantages, it is important to select the right concept depending on the application.

For TDS, the read-out signal spans the whole system bandwidth *B* at once. Therefore, a fast sampling has to be performed in the reader but also high measurement update rates can be achieved. Reflective delay line SAW sensors can be interrogated based on the pulse radar principle (Section 5.1). For resonant SAW sensors, there are two current architectures that directly determine the SAW’s resonance frequency from the response signal after a previous “charging”: digital frequency estimation and instantaneous frequency measurement, which will be presented in Section 5.2.1 and Section 5.2.2, respectively. The hybrid concept of FM/AM tracking loops (Section 5.2.3) also uses a pulsed excitation but requires, depending on the tracking strategy, at least two excitations at different and well-known frequencies per sensor value. Since the signal processing is clearly different from FDS, it is therefore classified as a TDS hybrid.

With FDS, the sensor’s frequency band is sampled in several steps. Thus, the baseband bandwidth can be rather low, resulting in a simpler and cheaper hardware design but also significantly longer interrogation times than with TDS. Using a frequency stepped continuous wave (FSCW) interrogation signal, the FDS technique is comparable to a one-port $S_{11}$ (monostatic) or two-port $S_{21}$ (bistatic) measurement on a vector network analyzer. When a linear frequency modulated continuous wave (FMCW) interrogation signal is used, the measurement is similar to common FMCW radar systems. Both excitation signals can be used for resonant as well as reflective delay line SAWs (Section 4.1). The hybrid concept S-FSCW will be shown in Section 4.2. It is a special form of FSCW and uses a pulsed interrogation with time-gating of the received signal. Since the signal processing is exactly the same as with FDS, it is classified as FDS hybrid.

## 4. Frequency Domain Sampling and FDS Hybrid Concepts

With FDS, the sensor’s frequency band is sampled in several steps and the sensor value is determined from the amplitude and phase/frequency differences between the transmitted and received signals. In contrast to pulse-based readout methods, this has the advantage of a relatively simple front-end structure, lower peak transmission power and reduced demands on the sampling rate. However, the maximum achievable sampling rate and, for most architectures, also the achievable dynamic range is considerably limited. The FDS technique can be used for both reflective delay lines as well as resonant SAW sensors; however, different signal processing steps are required depending on the sensor type. Furthermore, there are two possible interrogation signals: FMCW where a linear frequency-modulated transmit signal is used and FSCW where discrete frequencies are measured one after the other [43]. Both excitation signals, the underlying signal model and the sensor-specific signal processing steps will be presented and compared in Section 4.1. S-FSCW is a special form of FSCW for reflective delay lines only, where a time-gating of both the transmit (TX) and receive (RX) signals is used (Section 4.2).

### 4.1. Frequency Domain Sampling with FSCW/FSCW Interrogation

#### 4.1.1. Basic Operation Priciple

**Figure 3:**
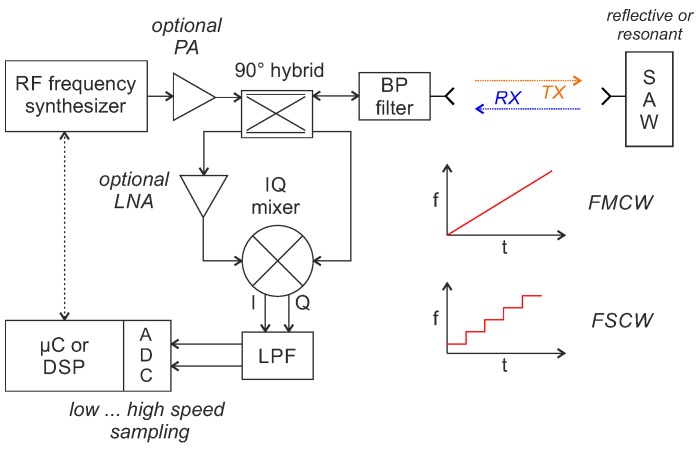
Block diagram of a monostatic reader concept for frequency domain sampling with frequency modulated continuous wave (FMCW) or frequency stepped continuous wave (FSCW) interrogation.

Frequency domain sampling with FMCW or FSCW interrogation is ultimately a distance measurement with a radar system that evaluates a single target (resonant SAW) or multi-target (reflective delay line SAW) scenario. The basic hardware structure, shown in Figure 3, looks accordingly similar to a continuous wave (CW) radar front end.

An RF frequency synthesizer generates the readout signal $s_{T}\left(t\right)$. For FMCW, this is a sinusoidal chirp, starting at $f_{st}$, with a linear increasing frequency:

(1) $$f\left(t\right)=f_{st}+kt.$$

The chirp rate k=B/T of the frequency sweep is defined by the effective bandwidth *B* and the sweep duration *T*. The transmitted signal then takes the form of [43]:

(2) $$s_{T}\left(t\right)=A_{t}\cos\left(2\pi \left(f_{st}+\frac{k}{2}t\right)t+\varphi_{0}\right)$$

with an amplitude $A_{t}$ and an arbitrary initial phase $\varphi_{0}$. FSCW interrogation also uses a frequency ramp but with discrete increased steps f[n] with (*n* = 0, 1, ..., *N* − 1) rather than continuously:

(3) $$f\left[n\right]=f_{st}+\frac{B}{N}\;n.$$

This way the transmitted signal takes the form [44]:

(4) $$s_{T}\left(t,n\right)=A_{t}\cos\left(2\pi f\left[n\right]t+\varphi_{0}\right).$$

The step duration $T_{step}$ of each step *n* must be long enough to ensure that both amplitude and phase of the signal reflected from the SAW are fully settled (also in the acoustic). Accordingly, the round trip delay time (RTDT) τ=2d/c must be significantly shorter than $T_{step}$.

Depending on the applications specific requirements, e.g., accuracy, measurement speed and cost, frequency synthesis can be achieved by a simple voltage-controlled oscillator (VCO), a stabilized phase locked loop (PLL), a direct digital synthesis (DDS) or a combination of those. Since the linearity of the (continuous or stepped) frequency ramp is of crucial importance for the later measurement accuracy [45], a lot of research has been carried out to generate highly linear ramps [44,46,47,48].

The transmit signal is typically amplified by an optional power amplifier (PA) before it is fed to the antenna via a 90${}^{\circ }$ hybrid coupler and bandpass (BP) filter. Instead of the 90${}^{\circ }$ hybrid, other concepts to separate the TX and RX signals, such as a directional coupler, circulator or bi-static approach, can be used too [49]. The RF signal is received by the antenna at the SAW, converted into an acoustic surface wave by the IDT and reflected back in accordance with the characteristics of the SAW. The acoustic wave at the IDT is then converted again into an electromagnetic wave and scattered back to the reader. There, it is received, amplified with an (optional) low noise amplifier (LNA) and finally down-converted with an IQ mixer that gets a part of the transmit signal as local oscillator (LO) signal. The baseband-signal is then low-pass filtered (LPF), amplified and finally digitized, typically with a micro-controller or digital signal processor (DSP). From the measured amplitudes and phase/frequency differences, it is then possible to calculate the properties of the sensors.

#### 4.1.2. Signal Model

It has been shown that it is possible to derive a valid signal model that describes the digitized baseband signal x[n], for both excitation signals as well as sensor types, by [43]:

(5) $$x\left[n\right]=\sum_{i=1}^{p}A_{i}\cos\left(2\pi \psi_{i}n+\varphi_{i}+\varphi_{ref,i}\right)+v\left[n\right]$$

with

(6) $$\psi_{i}=kT_{s}\tau_{i}=\frac{B}{N}\tau_{i},$$

where $A_{i}$$\psi_{i}$ and $\varphi_{i}$ represent the amplitude, normalized intermediate frequency (IF) and phase offset corresponding to the *i*th target. $\varphi_{ref,i}$ refers to an additional phase offset depending on the targets reflection properties and v[n] models additive white Gaussian noise, with zero mean and variance $\sigma^{2}$ [43]. The symbol *n* is the sample index with *N* total samples acquired by the analog-to-digital converter (ADC). For reflective delay lines, *p* equals the number of reflectors in the SAW sensors, whereas, for resonators, p=1. For frequency evaluation, the FSCW and FSCW signal models are equivalent, just for the phase evaluation, there is a minor difference [50]. For an FMCW interrogation, the phase offset $\varphi_{i}$ is calculated with:

(7) $$\varphi_{i,FMCW}=2\pi f_{st}\tau_{i}-\pi k\tau_{i}^{2},$$

whereas, for FSCW, it is:

(8) $$\varphi_{i,FSCW}=2\pi f_{st}\tau_{i}.$$

In addition, if, according to the signal model, the received signals contain the same information for FSCW and FMCW excitation, the representation is somewhat different: in the case of FSCW excitation, the received signal can be illustrated as discrete samples (I[f],Q[f]) in the spectrum. With FMCW excitation, on the other hand, there is a time-sampled signal (I[t],Q[t]): the transmitted frequency chirp, folded with the impulse response of the sensor, and mixed down with itself.

#### 4.1.3. Signal Processing for Reflective Delay Line Sensors

**Figure 4:**
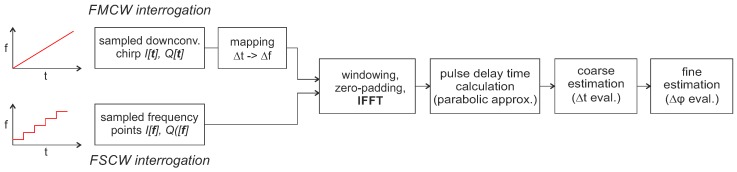
Signal processing flow chart for frequency domain sampling with reflective delay line SAW sensors.

In the case of reflective delay line sensors, the digitized baseband signal x[n] consists of a superimposition of the response signals of each individual reflector. The aim of the signal is now to determine the underlying impulse response of the sensor and thus the temporal distance between the individual reflectors. A flow chart of the typical signal processing steps is depicted in Figure 4.

After a suitable windowing and zero-padding the baseband data I[f],Q[f] measured with FSCW is transformed into the time domain by inverse fast Fourier transformation (IFFT). With FMCW excitation, the sampled baseband signal I[t],Q[t] is firstly mapped with Δt -> Δf using the chirp rate *k* and the ADC sampling rate and then further processing runs in the same way. At first glance, this may look different from the usual FMCW radar signal processing but is effectively the same: the fast Fourier transform (FFT) could also be calculated first and then a mapping from Δf to Δt could be performed since the FFT and IFFT are mathematically identical except for a normalization and the sign. In the time-domain, the coarse pulse delay time is then calculated via a parabolic approximation of the amplitude response [51]. In coherent systems, the time difference can then be further determined with much higher accuracy using a subsequent phase evaluation [23]:

(9) $$\varphi \left[t\right]=\arctan\left(\frac{Q[t])}{I[t])}\right).$$

With today’s systems, this yields accuracies of up to 1500 times higher than only amplitude evaluation [52]. However, ambiguities occur when the phase shift exceeds 360${}^{\circ }$ [53]. This is typically circumvented by placing multiple reflectors at known points and by evaluating all their phase differences to resolve the ambiguities [54]. The shown approach (Figure 4) is the simplest way to extract RTDT information from the measured data. Much more detailed work has been done, e.g., on optimizing window functions [55], taking material properties into account [56] or using model-based evaluation [57,58] to increase the accuracy.

#### 4.1.4. Signal Processing for Resonant SAW Sensors

**Figure 5:**
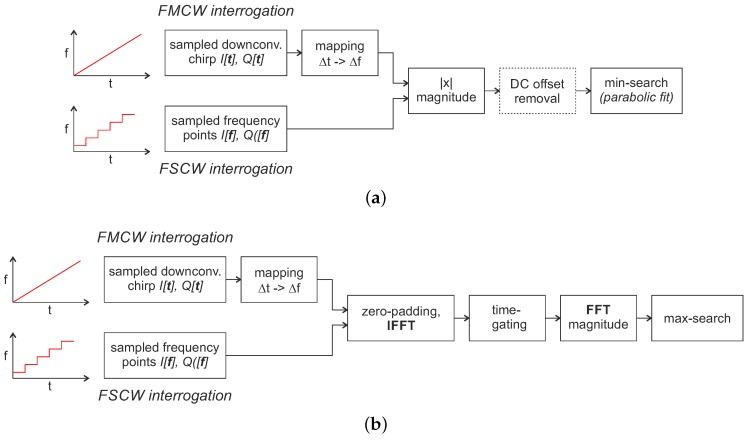
Signal processing flow chart for frequency domain sampling with resonant SAW sensors. (**a**) direct calculation of the resonance frequency from the sampled frequency points; (**b**) calculation of the resonance frequency using additional software time-gating to mask static reflections of the environment as well as mismatches and crosstalk within the front end.

The aim of signal processing for resonant SAW sensors is to determine its resonance frequency $f_{0}$. A flow chart of two possible approaches is depicted in Figure 5.

In the simplest case, the resonant frequency can be directly obtained from the magnitude of sampled frequency points, if necessary with a parabolic approximation to increase the resolution, as shown in Figure 5a. The algorithm searches for the lowest received amplitude, as the SAW has the best matching there and reflects the least of the excitation signal. This works well as long as the SAW is close enough to the reader and as long as there are only few static reflections in the environment and low mismatches and crosstalk in the front end. If the path attenuation or non-idealities increase, it becomes more and more difficult to detect the SAW in the received signal. An identified DC offset could be subtracted from all measured values, but this is not ideal since it is actually frequency-dependent, which is not taken into account in this algorithm. The second approach, shown in Figure 5b, uses a software time-gating to suppress the frequency-dependent but time static parasitic influences of the environment and frontend and can thus increase the sensitivity. To do so, the signal is transferred to the time domain via IFFT after an appropriate zero-padding to increase the resolution. There, all static influences are cut out by a time-gating. Since the SAW has a long response time due to its high quality factor, its signal is only minimally reduced in amplitude by this [59]. Then, the signal is transferred back into the frequency domain by calculating the FFT. The highest value in magnitude now corresponds to the resonance frequency of the SAW.

### 4.2. Frequency Domain Sampling with S-FSCW Interrogation

**Figure 6:**
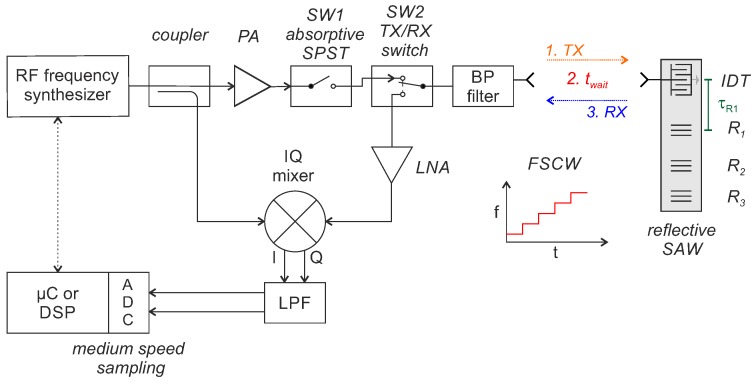
Block diagram of a monostatic reader concept using switched frequency stepped continuous wave interrogation for reflective delay line SAWs.

A major disadvantage of the FSCW/FMCW architectures presented so far is that they transmit and receive simultaneously and are thus considerably limited in the dynamic range. To avoid this, an Austrian research group presented a hybrid S-FSCW reader concept for reflective delay line SAW, firstly in 2004 [60]. The technique is a special form of FSCW interrogation but uses at least two additional high-speed RF switches (*SW1*, *SW2*) with high isolation to realize a hardware time-gating on both TX and RX signals. This way interfering reflections of the environment and mismatch in the front end can be suppressed so that a further amplification of the received signal, that would have previously saturated the LNA, is now possible [60]. This can be done because the response of a reflective delay line SAW has usually a delay of several 100 ns due to the slow propagation velocity of the surface wave on the piezoelectric substrate. A block diagram of the reader architecture is shown in Figure 6.

During the excitation phase (1. TX), SW1 is closed and SW2 is in TX position. The receiver is thus isolated from the high-power transmission signal. After the system and the acoustics have been completely settled, the TX signal is switched off first (SW1 open). Then, a short wait time is introduced (2. $t_{wait}$) in which the strong reflections of the immediate environment decay before, in the third step (3. RX), SW2 is switched to RX to receive the sensor response. In a further enhancement, the coupler is also replaced by an RF switch, toggling between the mixer LO port and the reader transmit path in order to fully use the synthesizer’s source power (so-called “double-switched FSCW” architecture [61]). The concept increases the signal to noise ratio (SNR) of the received signal due to the higher amplification of the RF signal and better utilization of the ADCs dynamic range. However, the maximum measurement time is then limited to $t_{m}=2\tau_{R1}-t_{wait}$, with $\tau_{R1}$ being the RTDT of the first reflector inside the SAW and thus an accordingly high enough base bandwidth must be used. The reader concept was also presented with four parallel receivers as multiple input multiple output (MIMO) system [62,63]. With the digital beamforming, it is then possible to realize an angle estimation and to separate several sensors at the same distance but with different angles.

S-FSCW can also be used for resonant SAW sensors. Instead of the IQ-mixer, a simple power detector is used for the received signal or alternatively it is mixed down with itself to determine the magnitude of received power. The excitation is then pulsed as with TDS systems (Section 5.2), but the signal processing is identical to an FSCW interrogation. Since the static reflections are already eliminated due to the hardware time-gating of the received signal, the resonant frequency can be determined directly as shown in Figure 5a.

### 4.3. State of the Art and Discussion for Frequency Domain Sampling and FDS Hybrids

Although all three architectures are based on the same concept of frequency domain sampling, each has its specific advantages and disadvantages in terms of dynamic range, update rate, measurement accuracy, and system costs. FMCW and FSCW are somewhat limited in their dynamic range as they have to transmit and receive simultaneously. S-FSCW can mitigate this disadvantage, by using a front end architecture similar to TDS, but at the expense of a mandatory higher baseband bandwidth. With FSCW and FMCW, the baseband bandwidth could be theoretically chosen as arbitrarily narrow to increase the measurement precision. In practice, however, this is not feasible because a single measurement would then take an extremely long time and no sufficiently high update rates could be achieved. Generally, (S-)FSCW readers are slower than FMCW readers because many individual frequency points have to be measured one after the other before a sensor value can be calculated. With FMCW, one frequency chip is sufficient to determine the sensor value. To do this, however, a frequency synthesizer is required that generates linear continuous frequency ramps. The reader costs then depend strongly on the ramp duration and required ramp linearity. Relatively cheap synthesizers can be used, which consequently only provide a limited update rate and measurement accuracy, or extremely fast chirps can be generated, e.g., via DDS and with a digital pre-distortion for maximum linearity [64]. This, however, requires significantly higher hardware effort. The stepped frequency ramp at FSCW is slower but has the advantage that it can be generated more cost-efficiently. The S-FSCW architecture is a little more costly due to the additional switches and higher baseband bandwidth but is still less complex than a typical FMCW implementation.

A direct comparison of the “real world performance” of different readers is difficult for various reasons: first of all, the quality of the measurement results does not only depend on the reader itself but is also strongly affected by the sensor properties (such as quality factor, matching, insertion loss, parasitic modes, hysteresis, sensitivity as well as cross-sensitives and others). Furthermore, depending on the measurement application, assembly and connection technology is still decisive. Last but not least, influences from the environment, such as multiple reflections, or the interface, when using, e.g., a rotational coupler, make a fair comparison even more difficult. Nevertheless, the realized FDS SAW reader systems presented so far [49,61,62,63,64,65,66,67,68,69,70] show exactly the previously discussed architecture-specific advantages and limitations.

A detailed comparison of a FSCW, S-FSCW and FMCW reader in the same environment and with the same reflective delay line SAW sensors in the 2.4 GHz industrial, scientific and medical (ISM) frequency band is given in [64]. The FSCW reader generated a stepped frequency ramp with a PLL-based frequency synthesizer and measures about 600 individual frequency points in the ISM frequency band. This requires a total of 125 ms, allowing a maximum of only eight measurements per second. The S-FSCW approach is a little faster here, since the measurement time per frequency point is limited by the architecture. A total of 75 ms is required to cover the ISM band so that thirteen measurements per second are possible. The FMCW reader is equipped with a fast DDS frequency synthesis that generates a chirp, covering the whole 2.4 GHz frequency band, in only 100 µs. This allows very high measurement rates to be achieved, but at significantly higher hardware costs: in addition to the DDS-based frequency synthesis, a 10 MSa/s ADC with 16-bit resolution is used for data acquisition and a fast DSP (TI TMS320C6713) is required to process them [67]. In a single shot measurement, the precision of the phase measurement was almost equal for the FSCW and S-FSCW reader and approximately five times higher for the FMCW reader [64]. However, if equal measurement times are considered, so that each reader can use different numbers of averaging, the results changed: the precision of the S-FSCW architecture was reported slightly lower than with FSCW and also the FMCW reader could improve with the standard deviation of the precision being only twice as high compared to the S-FSCW [64] reader.

## 5. Time Domain Sampling and TDS Hybrid Concepts

With TDS, the sensor’s whole bandwidth is covered at once. Therefore, compared to FDS, a faster sampling has to be performed in the reader but also higher measurement update rates can be achieved. Furthermore, due to the time multiplexing between TX and RX, it is possible to realize systems with a high dynamic range.

### 5.1. Time Domain Sampling for Reflective Delay Line Sensors: Pulse Radar Interrogation

**Figure 7:**
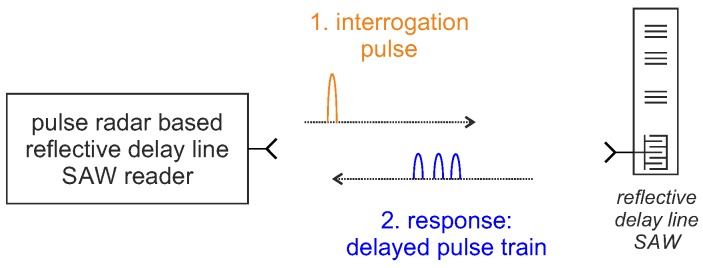
Schematic drawing of a reflective delay line SAW interrogation with a pulse radar based SAW reader.

The first reader generation for reflective delay line SAWs was based on the pulse radar principle. A schematic drawing of the readout process is shown in Figure 7. The concept is quite simple, but the challenge lies in the implementation of the required broadband receiver.

A fixed frequency transmitter with fast RF switches generates a short RF burst, usually only a few tens of nanoseconds [23,29], which is transmitted to the sensor. There, it is (partially) reflected by the individual reflectors and leads to a train of delayed non-overlapping pulses as response signal. These are down-converted to zero-IF, either with a simple power detector that can only evaluate the magnitude, or with an IQ mixer for additional phase evaluation. The latter is more complex, since a coherent receiver is required, but it allows significant higher measurement resolution. The major advantage of this architecture is that highly dynamic measurements are possible due to the short interrogation times [71]. However, the mandatory high bandwidth is also its huge disadvantage: the architecture is too costly and complex due to the necessary fast sampling and switching circuitry [72] and the required broadband excitation is not always compatible with the strict ISM band limits, especially not in the limited 868 MHz frequency band. Some prototypes have been built and evaluated [26,29,59,73,74,75,76]; however, this principle is only used in absolute exceptions when the very highest sampling rates are required for reflective delay line SAWs. A measurement update rate up to 250 kSa/s, which was only limited by the acoustic sensor design but not by the detection bandwidth, could be demonstrated in [76], but with a high hardware effort using 40 ns transmit pulses and a real-time field-programmable gate array (FPGA) signal processing. Almost all of today’s readers for reflective delay line sensors use one of the FDS or hybrid architectures, shown in Section 4, which are easier and more economical to implement. However, this could change in the future when the progressive integration of RF chips, software-defined radio solutions and fast ADCs offer more economical implementation options. A further interesting approach to reduce the currently high demands on the ADC is stroboscopic sampling [77,78], where the impulse response of the SAW is scanned by several excitations. This results in a higher measurement time per SAW and increased SNR because only a part of the received energy is used per measurement. However, due to the shifted sampling over several response signals, a considerably cheaper ADC can be used.

### 5.2. Time Domain Sampling for Resonant SAW Sensors

**Figure 8:**
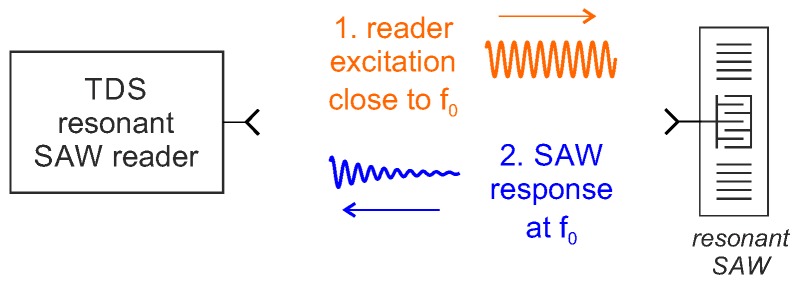
Block diagram of the reader concept for time domain sampling with digital frequency estimation.

Compared to reflective delay line SAWs, resonators have a lower insertion loss, higher quality factor and correspondingly a longer response time and require a lower bandwidth for a pulsed interrogation [38]. Therefore, time domain sampling is much more interesting for this type of sensor, especially for applications such as torque measurements on rotating shafts [79] which, in contrast to temperature measurements, require highly dynamic readers. A schematic drawing of a TDS interrogation process of a resonant SAW is shown in Figure 8.

In the first step, the resonator is “charged” by the reader with a CW excitation signal, mostly, but not always, as close as possible to the resonance frequency $f_{0}$ of the resonator. The excitation signal is received by the antenna of the SAW and the IDT connected to it converts part of the electromagnetic energy into mechanical vibration energy in the form of standing surface acoustic waves. Due to the time-gated excitation signal, a sine multiplied by a rectangle in the time domain results in a sinc in the frequency domain, an excitation close to the main resonance also has spectral components at $f_{0}$. After the excitation signal is quickly switched off, the IDT converts part of the mechanical energy back into an electromagnetic signal. Since the SAW is a purely passive linear device, the returned signal spectrum is the product of the emitted pulse spectrum and the SAW transfer function resulting in the maximum spectral component at $f_{0}$. This signal, which decays exponentially in amplitude, is then transmitted by the antenna, received by the reader and processed. The time required for charging (or discharging) depends on the quality factor of the loaded resonator $Q_{SAW}$ and its frequency $f_{0}$, which determine the time constant $\tau_{SAW}$:

(10) $$\tau_{SAW}=\frac{Q_{SAW}}{\pi \cdot f_{0}}.$$

When the excitation signal is switched off at t=0, the amplitude of the response signal of the SAW can then be calculated:

(11) $$A\left(t\right)=A_{max}\cdot e^{-t/\tau_{SAW}},$$

where $A_{max}$ is the maximum amplitude of the response signal, depending on the absorbed energy during the previous excitation step. The absorbed energy is further dependent on the received power, excitation time as well as mismatches and internal losses of the SAW. Commonly used excitation times for TDS sampling SAW readers are between $3\tau_{SAW}$ to $5\tau_{SAW}$ to enable charging of the SAW between 95% to 99.3% of its asymptotic value. For resonators produced with current processes, this means an excitation time of between just a few µs (resonator at 2.4 GHz with $Q_{SAW}$ = 2300) up to 50 µs (resonator at 433 MHz with a qualify factor of 13.300). The measurement time of the reader then depends on the dynamic range of the receiver and is usually of a similar duration.

Several different reader architectures for TDS of resonant SAW sensors have been developed up to now to evaluate the SAW’s response signal. Pohl et al. showed a relatively cheap reader design based on the use of a gated PLL in the transceiver in 1998 [80]. The sensor was interrogated with a pulsed excitation to stabilize the PLL regularly with its time-gated response signal. Thus, a frequency counter with a long integration time could still be used to determine the resonance frequency. However, the concept has one serious disadvantage: it requires one PLL for each sensor to be read. Modern force/torque measurement systems typically use at least three resonators [42], sometimes even five [81], so the hardware effort would increase significantly with this architecture. Today, three TDS architectures for resonant SAW sensors are still active in development. DFE, which will be presented in Section 5.2.1, instantaneous frequency measurement (IFM) (Section 5.2.2) and the TDS hybrid with pulsed FM/AM tracking loops (Section 5.2.3).

#### 5.2.1. Digital Frequency Estimation (DFE)

**Figure 9:**
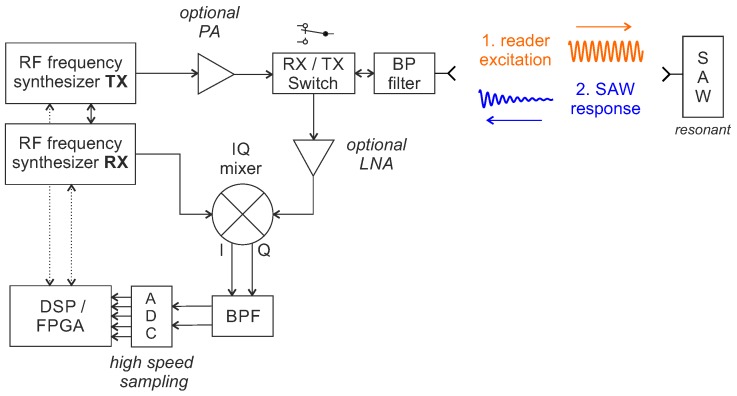
Block diagram of the reader concept for time domain sampling with digital frequency estimation.

The DFE reader architecture is based on the idea of algorithmically determining the frequency of the sensor’s down-converted and digitized response signal. A block diagram of the reader concept is depicted in Figure 9.

The TX RF frequency synthesizer, which can be implemented as a simple PLL, generates the readout signal: a CW excitation pulse that should be as close as possible to the resonance frequency $f_{0}$ of the sensor to be measured in order to transfer as much energy as possible. Depending on the application, the excitation signal can be amplified via an optional PA and is then transmitted via the antenna after passing an RX/TX switch. When the resonator has settled or a sufficiently long time (several $\tau_{SAW}$), the reader switches quickly from transmitting to receiving. The exponentially decaying received signal is amplified via an LNA and then down-converted to an IF with the RX synthesizer as LO. First, DFE designs used a two-stage heterodyne receiver [37], but, with today’s high quality integrated IQ mixers, a single stage is sufficient. In principle, the received signal could also be mixed down to (quasi) zero-IF with a part of the transmitted signal. This would eliminate the required second synthesizer; however, utilizing the whole dynamic range of the ADCs would be more difficult and DC offsets could limit the sensitivity of the receiver. For these reasons, IF frequencies between 700 kHz and 6 MHz are used in previous demonstrators [39,82,83,84].

The baseband signal is further amplified, band-pass or low-pass filtered and finally digitized by an ADC. Since the response signal is only a few microseconds long, depending on the frequency and quality factor of the interrogated SAW, a fast ADC is required to acquire sufficient samples for the later frequency estimation. For free space applications, a time-gating is usually applied to suppress echoes from the environment [59]. In general, the resonance frequency of the sensor can be determined from a single response signal. However, if no (such) high measurement update rate is required, a coherent averaging of several received signals is often carried out at this point to enhance the measurement precision [85]. When the measurement signal is superimposed with additive white Gaussian noise, the SNR of the result can be improved by $\sqrt{N_{a}}$ when $N_{a}$ signals are coherently accumulated. Then, the frequency of the digitized signal is evaluated, either by discrete Fourier transform and with zero-padding and/or parabolic interpolation [84,86] or by other frequency estimation algorithms that have recently received increased interest from the research community [87,88,89,90,91]. From this result and with knowledge of the excitation frequency, the resonant frequency $f_{0}$ of the SAW can be derived.

The architecture has the great advantage that a single excitation is in general sufficient to obtain a sensor value and thus the measuring time is theoretically limited only by the sensor itself. In practical implementations, however, the computational intensive digital frequency calculation is usually the bottleneck and fast DSPs or FPGAs must be used if high measurement update rates are to be achieved. Furthermore, if not a single but several sensors are read out, the lock time of the synthesizer plays a role, since it must jump to the resonance of the individual SAWs every time. The receiver phase noise is a further parameter as it affects the measurement precision. Especially in free space applications, with sensor distances equal or less 1 m, it plays the dominant role [85].

When really fast measurement update rates are required and the algorithms used for frequency calculation have a sufficient frequency resolution, several sensors can in principle also be excited and read simultaneously, as shown by Kalinin et al. in 2012 [92]. However, care must be taken to reduce the mutual influence of the SAWs and to design the baseband properly to avoid errors due to aliasing and intermodulation products that could occur due to simultaneous excitation.

#### 5.2.2. Instantaneous Frequency Measurement (IFM)

**Figure 10:**
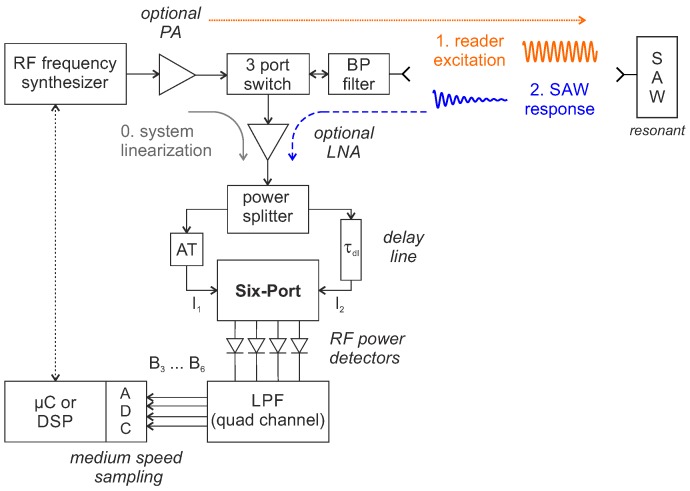
Block diagram of the reader concept for time domain sampling with instantaneous frequency measurement.

Recently, a further reader concept for resonant SAW sensors was proposed: it is based on time domain sampling with an (analog) instantaneous frequency measurement of the sensor’s response signal using a six-port microwave interferometer and a delay line [93,94,95]. The most notable advantage to other TDS architectures is that it measures the resonance frequency directly from a single interrogation but without the need to calculate the arithmetic-intensive FFT or other frequency estimation algorithms. The IFM technique was already developed in the 1950s for military applications like radio reconnaissance and has continuously emerged for radar warning purposes and as electronic warfare receivers [96,97,98]. It is still used today in defense applications for real-time frequency identification of unknown signals over a very wide bandwidth but typically in a fully digital implementation [99]. However, especially the analogue approach, combined with digital compensation of non-idealities, is very promising for industrial sensor technology, such as wireless SAW readers, as it provides high measurement update rates at comparably low hardware costs [95]. Although the IFM concept has been used in military receivers since the 1950s, it was the work of Engen and Hoer in the 1970s [100,101] that made the six-port well-known for other civil applications. Today, six-port and multi-port structures are used in a wide variety of metrology and communication applications ranging from vector network analysis, distance, vibration and angle of arrival measurements to direct conversion receivers with gigabit data rates [102,103,104,105,106,107]. Figure 10 shows a block diagram of the IFM reader concept for resonant SAW sensors.

An RF frequency synthesizer generates the narrow-band excitation pulse, as close as possible to the resonant frequency of the SAW. This signal is amplified by an optional PA and passes a switch and BP filter before it is radiated by the antenna to the SAW. After a sufficiently long excitation time, the switch is changed from transmit to receive mode and the decaying response signal of the SAW is received. This signal is divided into two parts. One part is fed directly to the first input port of the six-port structure, optionally through a variable attenuator (AT) to adjust the power. The second part passes a delay line with the time delay $\tau_{dl}$ before being fed to the second input port $P_{2}$ of the six-port structure. This leads to a frequency-dependent relative phase shift Δφ between the two signals ($I_{1}$, $I_{2}$) of:

(12) $$\Delta \varphi =2\pi f\tau_{dl}.$$

**Figure 11:**
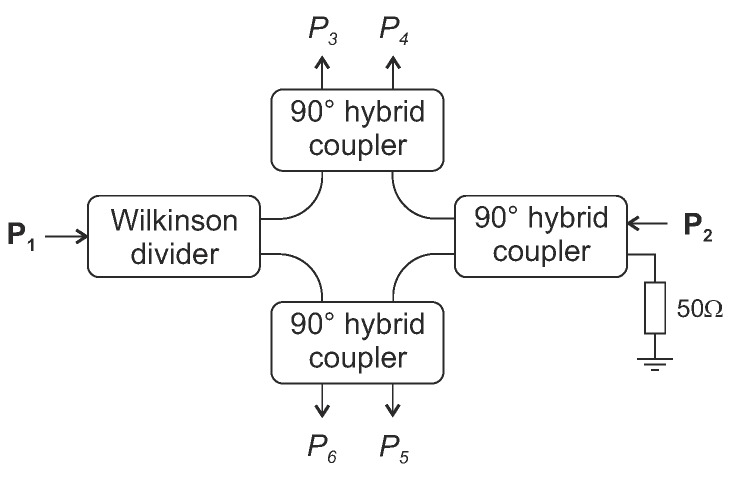
Passive structure of a six-port interferometer formed by a Wilkinson power divider and three 90° hybrid couplers.

The time $\tau_{dl}$ depends on both the geometrical length $d_{g}$ of the delay line as well as on the effective relative permittivity $\epsilon_{r,eff}$ of the used material and conductor structure and can be calculated by

(13) $$\tau_{dl}=\frac{d_{g}\cdot \sqrt{\epsilon_{r,eff}}}{c_{0}},$$

with $c_{0}$ denoting the speed of light in vacuum. The resulting phase difference is then evaluated by the six-port interferometer, which can be realized as a passive structure formed by a Wilkinson power divider, three 90° hybrid couplers and a matched termination, as shown in Figure 11 [104].

Within the six-port structure, the two input signals are superimposed under four relative phase differences of 0, π/2, π and 3π/2 leading to four output signals ($P_{3}$ ... $P_{6}$). Using RF power detectors, a direct conversion to baseband can be realized and the four Equations (14)–(17) describe then the baseband voltages $B_{3}$ ... $B_{6}$ whose amplitudes are dependent on the phase difference Δφ of the RF input signals $I_{1}$ and $I_{2}$:

(14) $$\begin{array}{c} B_{3}={\left|P_{3}\right|}^{2}=\frac{1}{4}\left(P_{1}+P_{2}+2\sqrt{P_{1}P_{2}}\sin\left(\Delta \varphi \right)\right), \end{array}$$

(15) $$\begin{array}{c} B_{4}={\left|P_{4}\right|}^{2}=\frac{1}{4}\left(P_{1}+P_{2}-2\sqrt{P_{1}P_{2}}\sin\left(\Delta \varphi \right)\right), \end{array}$$

(16) $$\begin{array}{c} B_{5}={\left|P_{5}\right|}^{2}=\frac{1}{4}\left(P_{1}+P_{2}+2\sqrt{P_{1}P_{2}}\cos\left(\Delta \varphi \right)\right), \end{array}$$

(17) $$\begin{array}{c} B_{6}={\left|P_{6}\right|}^{2}=\frac{1}{4}\left(P_{1}+P_{2}-2\sqrt{P_{1}P_{2}}\cos\left(\Delta \varphi \right)\right). \end{array}$$

These baseband voltages are then lowpass-filtered and amplified before they are digitized by, usually four simultaneously sampling, ADCs [108]. A single multiplexed or dual-ADC could also be used but with higher demands on the sampling rate and additionally necessary signal processing to compensate the phase error induced by the switching [109]. In general, a single simultaneous sample (on all four channels) is sufficient to determine the response frequency of the SAW. Accordingly, the requirements for the ADC sampling rate are comparable to FM/AM tracking loops and significantly lower than with DFE. The four digitized baseband voltages can be interpreted as a complex valued number $\underline{z}$ in differential representation:

(18) $$\underline{z}=\left(B_{5}-B_{6}\right)+j\left(B_{3}-B_{4}\right)\;.$$

The argument of $\underline{z}$ is equal to the phase difference between the RF input signals $I_{1}$ and $I_{2}$ and can be calculated by using, e.g., the atan2 function:

(19) $$\Delta \varphi =\arg\left(\underline{z}\right)=\mathrm{atan}2\left(\frac{B_{3}-B_{4}}{B_{5}-B_{6}}\right).$$

Using this value and with the knowledge of the exact length $\tau_{dl}$ of the delay line, the originally unknown input frequency can now be calculated by transforming Equation (12) to:

(20) $$\tilde{f}=\frac{\Delta \varphi }{2\pi \cdot \tau_{dl}}.\;$$

Since the concept can only measure a phase difference and not an absolute phase, this calculation becomes ambiguous when the effective length of the delay line is longer than the wavelength of the highest frequency to be measured [98]. However, it has been shown that this is not a practical limitation for SAW readers as only a very small unambiguous measurement range is generally required for resonant sensors [95]. The above formulas are all based on idealized conditions such as perfectly matched components with no imbalances and completely linear power detectors that cannot be achieved in reality due to manufacturing tolerances, temperature influences and aging. To compensate for these hardware non-idealities, calibration and linearization concepts similar to those already established with other six-port systems can be used [110,111,112,113,114,115]. The alternative concept of a complete system linearization with one or more well-known signals of the frequency synthesizer, shortly before the SAW response signal is measured [116,117] looks promising as it derives each measurement from the frequency accuracy of the reference crystal in the synthesizer. This is particularly necessary in practical applications, as the temperature drift of the delay line would otherwise cause the measuring accuracy to degrade. When a SAW resonator at 2.4 GHz, with a maximum frequency deviation of 1 MHz, is to be determined with an accuracy of 1% of the measurand, an RF frequency accuracy of approximately four parts per million (ppm) is required. Differential measurement can reduce this requirement [95], but it still remains a challenge. Possible forms of implementation for delay lines, e.g., as SMA cable [118], PCB-based [119] or as SAW delay line [120] all have a temperature sensitivity, typically in the two-digit ppm range. A differential measurement reduces the influence considerably, but nevertheless even minor temperature fluctuations could degrade the measurement accuracy. However, since temperature changes slowly, the actual length of the delay line can be determined by (one or more) reference measurements with known frequencies directly before the measurement of a SAW to completely compensate this influence.

#### 5.2.3. Pulsed FM/AM Tracking Loops

The first idea of using the FM to AM conversion property of resonant SAW sensors for their wireless interrogation originated already more than 20 years ago in a patent from Anthony and Bryan Lonsdale [121]. In 1995–2002, Transense Technologies (Weston-on-the-Green, UK) developed and used a short-range reader based on this idea for electric power-assisted steering systems [122]. The architecture used a continuous excitation with frequency modulated signals around the resonance of the sensor, detected the first harmonic in the amplitude of the reflected signal and used automatic frequency control (AFC) loops to follow any changes [122]. With a modulation frequency $F_{mod}$ = 20 kHz and a frequency deviation of 20 kHz, a time constant of the AFC loop down below 0.6 ms was reached and correspondingly high measurement update rates could be realized. Due to the continuous excitation and many measured frequency points, this architecture can be clearly assigned to FDS. The original concept was later no longer pursued in favor of the DFE architecture, mainly due to the reader costs and difficulty to apply the same method for interrogation of three or even five resonators [39].

**Figure 12:**
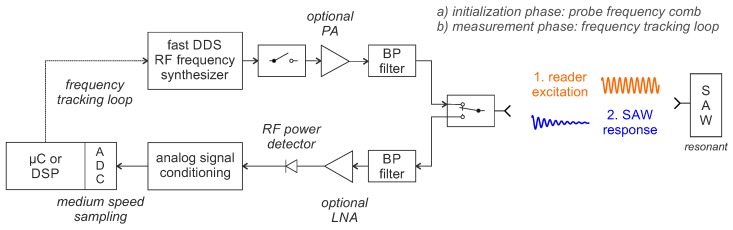
Block diagram of the reader concept for time domain sampling with pulsed frequency modulation (FM)/amplitude modulation (AM) tracking loops.

Nevertheless, the fundamental idea was picked up again by Friedt et. al. in 2010 [123] but with a modified architecture as TDS hybrid system. Advances in microelectronics and the development of fast DDS chips with small frequency increment made a pulsed readout and evaluation in FM/AM tracking loops possible and promising. In the proposed concept, a narrowband pulsed excitation and evaluation of the sensors response signal is used. With, depending on the tracking strategy, two, three, or several interrogations, the resonant frequency can be estimated based on the received amplitude differences of the individual response signals. A block diagram of the reader concept for pulsed FM/AM tracking loops is depicted in Figure 12.

A fast DDS-based RF frequency synthesizer is required to generate the excitation signal because it is crucial for the system concept to jump back and forth between several frequencies very quickly and accurately. The transmit signal passes a fast single pole, single throw RF switch, which is used for pulsing, and is amplified by an optional PA. After the resonator has settled for several $\tau_{SAW}$, the RF switch opens and the reader changes from TX to RX mode to measure the free oscillation radiated by the sensor. This gets received, amplified by an LNA and demodulated to baseband. In the simplest case, an RF power detector performs a direct conversion to baseband by measuring the RF power. Alternatively, IQ demodulators can also be used to improve the resistance to unwanted emitters [123]. The baseband signal is further amplified and band limited before it is digitized by an ADC. A single sample per response signal is sufficient, so that no very fast ADC is required as with the DFE method. In the first initialization phase of the reader, the sensor’s resonant frequency must be roughly determined. This is done by probing along a frequency comb and searching for the maximum response amplitude in the whole frequency range where the sensor could be [124]. Due to the large number of measuring points and FDS-based signal processing, the so-called “fixed comb” strategy [125] can then be classified as FDS hybrid with S-FSCW interrogation (Section 4.2).

In the subsequent TDS hybrid measurement phase, narrowband tracking loops follow the resonant frequency of the SAW. Depending on the strategy, these can require several pulsed FM interrogations around the SAW or only require two or three interrogated frequency points per sensor value with identifying the maximum received signal amplitude.

##### 5.2.3.1. Three-Point Interrogation Strategy

**Figure 13:**
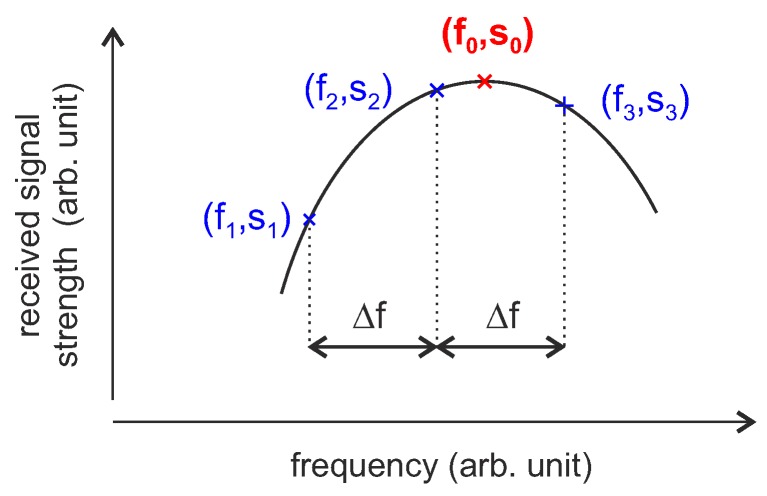
Pulsed FM/AM tracking loops with three-point interrogation strategy.

The first tracking strategy is shown in Figure 13. Using three interrogated frequency points, one must be above and one below the resonance frequency of the sensor, while the third one can be arbitrary. The frequencies should, however, have the same distance Δf to each other to simplify the subsequent signal processing.

From the measured amplitude values ($s_{1},s_{2},s_{3}$) at the frequency points ($f_{1},f_{2},f_{3}$), the resonant frequency $f_{0}$ of the sensor can then be calculated using a parabolic fit as an approximation of the Butterworth–van Dyke (BvD) response of the resonator [126]. With $f_{1}$, $f_{2}$ and $f_{3}$ equally spaced with Δf the calculation of $f_{0}$ simplifies to [123]:

(21) $$f_{0}=f_{2}+\frac{\Delta f}{2}\cdot \frac{s_{1}-s_{3}}{s_{1}+s_{3}-2\cdot s_{2}}.$$

The choice of Δf is thereby a trade-off between maximizing the amplitude differences $s_{3}-s_{2}$ and $s_{2}-s_{1}$ by choosing a large Δf and minimizing the error made by approximation with the 2nd order Taylor development by keeping Δf small enough [123]. Numerical simulations have shown that the error between the polynomial fit and the true resonance shape differ by less than ±1 % for [123]:

(22) $$\Delta f\leq \frac{f_{0}}{3\cdot Q}.$$

There are several options for the control loop that follows the SAW. Low noise and bias is observed when trying to minimize the error between $f_{2}$ and $f_{0}$ [123]. For this purpose, the fast DDS signal source is needed, which adapts the excitation frequencies from measurement to measurement.

##### 5.2.3.2. FM Interrogation Strategy

**Figure 14:**
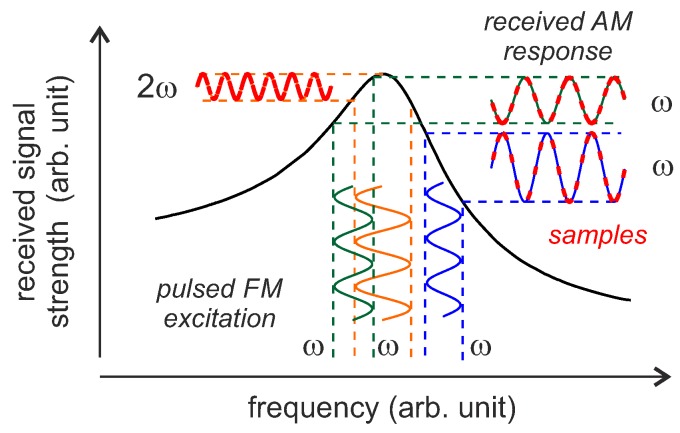
Pulsed FM/AM tracking loops with FM interrogation strategy and signed phase evaluation.

The FM tracking strategy uses the original idea of FM interrogation signals [122] but with a pulsed excitation and an additional phase offset evaluation between the FM transmission signal and the received signal [127,128]. This strategy requires several more measured frequency points per sensor value, which leads to a lower measurement update rate, but the measurement accuracy increases in return. The basic concept is shown in Figure 14.

The resonator is excited with a pulsed FM signal with the modulation frequency $\omega_{m}$ and then the amplitude of the response signal is evaluated. In order for the resonator to observe a quasi-static signal within a single measuring point, it is necessary that $\omega_{m}$ is not too high. It was proposed in [127] to use a modulation frequency less or equal to:

(23) $$\omega_{m}\leq \frac{1}{10}\cdot \frac{1}{2\tau_{SAW}}.$$

When the slope of the transfer function of the resonator is rising (left/green interrogation), the emitted FM signal is received as an amplitude modulated signal with $\omega_{m}$. At resonance frequency (middle/orange), the part of $\omega_{m}$ in the response signal disappears and only $2\omega_{m}$ remains. When the slope of the transfer function is negative (right/blue) again, an AM signal at $\omega_{m}$ is received but with an inverted phase. Since there is a smooth transition between these three states, the feedback control of the reader can now use a linear varying parameter (phase change around the resonance frequency) instead of trying to identify the maximum received power (as for the three-point and two-point interrogation strategy) [125]. For this purpose, the received signal is band-pass filtered at $\omega_{m}$ and the phase to the FM excitation signal is evaluated. For excitation to the left of the resonant frequency, there is a positive phase difference; for excitation exactly at the main resonance, the phase difference is zero and for excitation to the right of the resonant frequency, the phase difference becomes positive [128]. This results in a higher accuracy at the costs of longer interrogation times.

##### 5.2.3.3. Two-Point Interrogation Strategy

**Figure 15:**
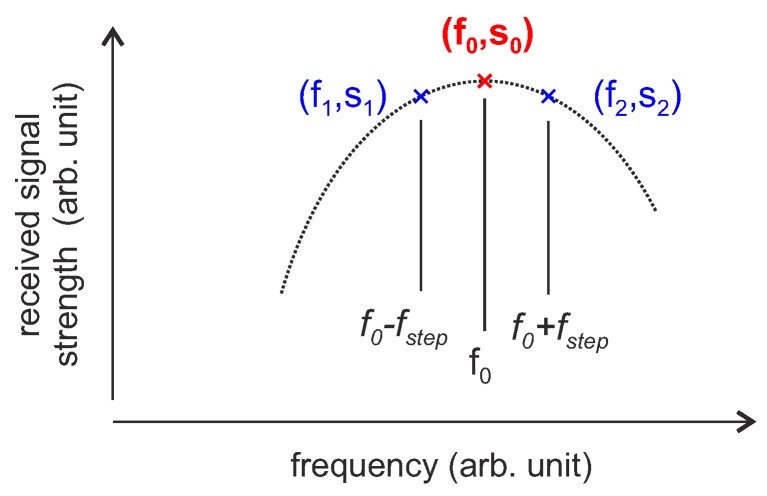
Pulsed FM/AM tracking loops with two-point interrogation strategy.

Based on the FM interrogation strategy, a further concept was investigated that requires only two excitation frequencies per sensor measurement [124]. The concept is based on the idea that it is sufficient to query only the two extreme values of the sinusoidal FM-sine wave while balancing the received power of the rising slope on one side of the resonance frequency with the falling slope on the other side. The two-point interrogation strategy is depicted in Figure 15.

The SAW sensor is probed at $f_{1}=f_{0}-f_{step}$ and $f_{2}=f_{0}+f_{step}$ and a tracking algorithm controls the interrogation frequencies in order to keep the amplitude of the response signal equal ($s_{1}=y\left(f_{0}-f_{step}\right)=s_{2}=y\left(f_{0}+f_{step}\right)$). As in the previous concept, $f_{step}$ must be selected carefully and should be below Δf (Equation (22)). This is quite a restriction for this as well as the three-point interrogation strategy. As each successive displacement of *f* must be less than $f_{step}$ in order not to loose the tracking [124], the maximum frequency of the measured variable $f_{m,max}$ is thereby limited to [92]:

(24) $$f_{m,max}=\frac{f_{tr}}{2\pi f_{m}t_{m}},$$

assuming full modulation of the sensor with a frequency variation $f_{m}$, at a tracking bandwidth $f_{tr}$ and the measurement time $t_{m}$ (two or three excitations including oscillation). When several sensors are measured consecutively, the maximum frequency is further reduced as the time interval between measurements of a single resonator increases.

### 5.3. State of the Art and Discussion for Time Domain Sampling and TDS Hybrids

The TDS reader architectures DFE and IFM can both determine the resonant frequency of an SAW resonator with only one excitation. This allows very high measurement update rates to be reached, which is particularly interesting for industrial measurement technology, e.g., for high-dynamic torque measurements. The main difference between the two approaches is the evaluation of the SAW response signal. With DFE, the frequency evaluation takes place in the digital domain. This is algorithmically most flexible and quite a lot of research is done in this area to achieve more efficient evaluation than with a pure discrete Fourier transformation. Furthermore, it is the only TDS architecture with which several sensors can be read simultaneously when a frequency estimation algorithm is used that can resolve multiple frequencies. However, DFE requires a fast and comparably expensive ADC to acquire enough samples for frequency estimation during a decay process and the still computational-intensive algorithms require fast DSPs or even FPGAs that make the reader even more costly or limit the effective maximum readout rate when slower devices with longer processing times are used. With IFM, the actual frequency determination is carried out in analog domain. The advantage is that the ADC can sample considerably slower than with DFE because generally a single sample per channel, simultaneously over four channels, is sufficient per readout of an SAW. In addition, no computationally complex algorithms are required since the frequency can be determined from the samples using simple trigonometry. However, the system must be regularly linearized during runtime in order to compensate for hardware non-idealities and temperature influences in situ. Furthermore, more attention must be paid to interference susceptibility, as the entire frequency measurement is mapped to a phase measurement. The hybrid concept with pulsed FM/AM tracking loops combines the FDS advantage of the low hardware effort with a fast TDS measurement. With different tracking strategies, either a high measurement update rate can be achieved (three- and two-point interrogation) or the focus can be placed on slower but more accurate measurements (FM interrogation). However, it must be ensured that the SAW does not leave the narrow-band tracking bandwidth during two consecutive measurements. In practical applications, this limits, despite the theoretically higher measurement update rate, the effective bandwidth of the measurand and is a considerable limitation for fast varying processes.

When considering the current state of the art, as with the FDS readers, it must be noted that the performance here also depends on the ambient conditions and the SAWs used so that the results can only be viewed in the overall context. For resonant SAWs, especially spurious modes [129] pose a challenge, since, through them, the actual mono-frequent response signal contains further spectral components. Depending on the position and characteristics, these can be difficult for the reader to separate and may affect the measurement accuracy. Therefore, there are also current research projects on optimizing SAW resonators to suppress their spurious modes directly in the sensor [130].

Several DFE-based readers have recently been published [83,89,92,131]. In [89], for example, a 14-bit ADC with sampling rate of 9 MSa/s is used to digitize the down-converted response signal of resonators at 428 MHz with a quality factor around 6000. The currently fastest DFE reader was published in [92] and provides a measurement update rate of 16 kHz for a differential measurement in the 433 MHz frequency band using a simultaneous excitation of two resonators. The measurement accuracy has been estimated at better than 1% and the precision (standard deviation of the random errors) was measured at 0.04%. In order to realize this, however, a high hardware effort is necessary and the reader requires two parallel RF frequency synthesizers, two DSPs for calculating the FFTs and an additional micro-controller to manage the whole system.

IFM is a very new architecture for which the first detailed investigations were published in [93,94,95]. Measurement times of 3 µs for the interrogation of a single resonator at 2.4 GHz SAW with a quality of approximately 2300 could be demonstrated and the precision in a differential measurement was approximately 1% [95]. However, an oscilloscope with a limited resolution was used to acquire the baseband signals and the measurement time was also significantly shorter than with readers in the 433 MHz ISM band.

Using pulsed FM/AM tracking loops, several demonstrators have also been published featuring the three-point interrogation [123], two-point interrogation [124] or FM interrogation [125,127,128] strategy. Up to 5 kHz for a single SAW resonator has been shown in [124] using the two-point interrogation strategy. Further experiments and evaluations have been made using 7 kHz sample rate for a single SAW sensing element and 3 kHz for differential sensing [132,133]. For FM interrogation, a significantly lower standard deviation was achieved, but the query time per sensor was then in the range of several tens of ms.

## 6. Overall Comparison and Discussion

As already presented and discussed with the individual sensor and reader architectures, all have their own advantages and disadvantages and it is important to find the right concept depending on the application. Reflective delay lines can combine identification with sensing and have the advantage that they can use TDMA, CDMA and OFC, compared to resonant sensors that can only employ FDMA for multiple access. Accordingly, they are particularly suitable for applications where many sensors with rather slow measured values, e.g., temperature, are to be read out. Additionally, with FDS, relatively inexpensive readers can be realized exactly for this application. The simplest and at the same time slowest architecture is FSCW, which measures several individual frequency points in the spectrum and calculates the impulse response from these. With the FMCW principle, generally higher measurement update rates can be achieved, but only with higher hardware effort, as fast linear frequency ramps are necessary. S-FSCW is a hybrid concept for reflective delay line SAWs where the dynamic range can be increased since no full duplex front end is required. The pulsed radar principle is currently not used in practical applications due to the high hardware costs but is the theoretically fastest possible concept when extreme update rates for reflective delay line SAWs are required.

Resonant SAWs have the benefit of lower insertion loss and a high quality factor and are particularly suited for TDS and TDS hybrid reader architectures that can achieve high measurement update rates. This makes them particularly interesting for industrial process monitoring, e.g., for determining force and torque on fast-rotating axes. Usually, RF rotation couplers are used in these scenarios for wireless interrogation, which have the advantage of a good signal transmission and higher robustness against external interference compared to a free space transmission. There are several possibilities for the realization of such couplers, e.g., based on electrically shorted transmission lines or open coplanar strip lines [134,135]. In general, care must be taken to minimize the parasitic frequency shift (known as “pulling effect”) caused by angle-dependent impedance variations of the coupling elements [135]. With DFE and IFM, the fastest measurements are possible because the resonance frequency of the sensor can be determined from a single measurement. In practical applications, however, the subsequent computationally complex signal processing that must be carried out on an embedded DSP and/or FPGA limits the measurement update rate at DFE. IFM and the hybrid concept of pulsed FM/AM tracking loops are significantly less computationally complex; however, in the first case, additional linearization steps are necessary and care must be taken that the sensor does not leave the narrow-band tracking loops in the latter case, which considerably reduces the effective usable bandwidth.

Since the sensors usually operate in freely available ISM bands, immunity to interference is an important issue for all architectures. If several SAW readers are used at the same time or when other transmitters such as WiFi or Bluetooth are active in the same frequency band, it is important to consider interference resolution strategies to minimize mutual impact [136]. However, there are also possibilities in the reader design to detect interferences [137], in order to reduce their influence by frequency hopping [138] or to avoid them by listen-before talk [139].

There are currently several companies that develop and distribute commercial SAW readers, e.g., CTR (Villach, Austria), IntelliSAW (Andover, MA, USA), Kongsberg Maritime (Kongsberg, Norway), Pro-micron GmbH & Co. KG (Kaufbeuren, Germany), RSSI GmbH (Geretsried, Germany), SAW COMPONENTS Dresden GmbH (Dresden, Germany), SENSeOR SAS (Valbonne, France), Sensor Technology Ltd (Banbury, UK), senTec-Elektronik (Ilmenau-Langewiesen, Germany), and Transense Technologies plc (Weston-on-the-Green, UK). However, public data sheets for their readers, if available at all, usually contain only very limited information such as the frequency range and the maximum sampling rate [140,141,142,143,144,145]. On the one hand, this may be due to the protection of their intellectual property. On the other hand, as already discussed, the performance also depends on the environment and the SAWs used, which makes it difficult to make a generally valid statement. In research, software defined radio (SDR) platforms are particularly popular because of their high flexibility and easy customization. The SDR hardware is of course much too expensive for large-scale commercial use, but new algorithms and interrogation strategies can be evaluated quickly and efficiently. In [146], a PXIe-based SAW interrogator has been presented that can operate in any frequency band between 85 MHz up to 6.6 GHz. Likewise, the popular universal software radio peripheral (USRP) platform widely used for research in wireless communication was programmed as the reader for OFC SAW sensor interrogation [147,148]. The front end of the USRP B200 platform can process RF signals between 70 MHz–6 GHz with an instantaneous bandwidth of 56 MHz.

## 7. Conclusions

This review paper presented the state of the art for wireless SAW readers and provided a guide to choose the appropriate sensor and reader architecture for each application. First, the basics of SAW sensor technology and the two types of SAW resonators and reflective SAW delay lines were presented. Then, the current architectures were introduced and an extended classification according to time-domain sampling, frequency-domain sampling and hybrid TDS/FDS architectures was suggested. Each architecture was studied in detail and discussed with regard to its specific advantages and disadvantages.

To sum it all up, SAW sensors are a promising technology with several unique features. Due to the purely passive and highly robust sensors, there are particularly promising applications in harsh industrial environments and exciting combinations of sensor technology together with radio identification. However, despite its outstanding technical features and many research activities in the last decades, SAW sensor technology is still a low-volume professional niche application, especially compared to the booming multi-billion dollar business of RF SAW filters. The authors see the reasons mainly in fabrication costs of reader devices, which are still clearly too high. This results in a classic chicken-egg problem: since the sensors are currently only used in small quantities, the costs for the readers are still relatively high. In addition, as long as the costs for the readers are still (too) high, the sensors will not be used more extensively. Thus, this is a very exciting question for the future if, when and by which technologies, a breakthrough will be achieved. On the one hand, the continuous progress of microelectronics and Moore’s law will automatically contribute to this. On the other hand, research on new reader architectures is also going on, as shown in this article, and is focusing more and more on reducing the costs per device. For really high quantities and low costs, there will be no way around monolithic integrated microwave circuits optimized for the needs of SAW sensors in the long run. Radar sensors can be seen here as a shining example that has already successfully taken this development. Just ten years ago, radar systems consisted of a large number of commercial of the shelf components and were comparatively bulky, heavy and expensive, similar to SAW readers today. However, thanks to highly integrated circuits, there are now complete microwave and mm-wave integrated radar front ends on a only few square millimeters space at prices of just a few dollars per unit. It will be exciting to see whether this breakthrough will also succeed with the SAW readers of a future generation.
